# Outcomes after primary and repeat thermal ablation of hepatocellular carcinoma with or without liver transplantation

**DOI:** 10.1007/s00330-021-08515-3

**Published:** 2022-02-08

**Authors:** Christiaan M. C. Serbanescu-Kele Apor de Zalán, Simeon J. S. Ruiter, Aad P. van den Berg, Jan Pieter Pennings, Koert P. de Jong

**Affiliations:** 1 Department of Hepato-Pancreatico-Biliary Surgery and Liver Transplantation, University Medical Centre Groningen, University of Groningen, Hanzeplein 1, 9713 GZ Groningen, The Netherlands; 2 Department of Gastroenterology, University Medical Centre Groningen, University of Groningen, Hanzeplein 1, 9713 GZ Groningen, The Netherlands; 3 Department of Radiology, University Medical Centre Groningen, University of Groningen, Hanzeplein 1, 9713 GZ Groningen, The Netherlands

**Keywords:** Carcinoma, Hepatocellular, Liver Neoplasms, Hyperthermia, Induced, Liver Transplantation, Neoplasm recurrence, Local

## Abstract

**Objectives:**

Thermal ablation (TA) is an established treatment for early HCC. There is a lack of data on the efficacy of repeated TA for recurrent HCC, resulting in uncertainty whether good oncologic outcomes can be obtained without performing orthotopic liver transplantation (OLTx). This study analyses outcomes after TA, with a special focus on repeat TA for recurrent HCC, either as a stand-alone therapy, or in relationship with OLTx.

**Methods:**

Data from a prospectively registered database on interventions for HCC in a tertiary hepatobiliary centre was completed with follow-up until December 2020. Outcomes studied were rate of recurrence after primary TA and after its repeat interventions, the occurrence of untreatable recurrence, OS and DSS after primary and repeat TA, and complications after TA. In cohorts matched for confounders, OSS and DSS were compared after TA with and without the intention to perform OLTx.

**Results:**

After TA, 100 patients (56·8%) developed recurrent HCC, of whom 76 (76·0%) underwent up to four repeat interventions. During follow-up, 76·7% of patients never developed a recurrence unamenable to repeat TA or OLTx. OS was comparable after primary TA and repeat TA. In matched cohorts, OS and DSS were comparable after TA with and without the intention to perform OLTx.

**Conclusions:**

We found TA to be an effective and repeatable therapy for primary and recurrent HCC. Most recurrences can be treated with curative intent. There are patients who do well with TA alone without ever undergoing OLTx.

**Key Points:**

• *Recurrent HCC after primary TA can often be treated effectively with repeat TA. Survival after repeat TA is comparable to primary TA*.

• *In matched cohorts, outcomes after TA with and without subsequent waitlisting for OLTx are comparable*.

• *There are patients who do well for many years with primary and repeat TA alone; some despite multiple recurrences.*

**Supplementary Information:**

The online version contains supplementary material available at 10.1007/s00330-021-08515-3.

## Introduction

Hepatocellular carcinoma (HCC) is a major cause of global morbidity and mortality [1], with an rising incidence in the Western world due to an increasing prevalence of steatohepatitis [2]. Staged according to the BCLC classification, only very early (single tumour, < 2 cm) and early HCC (up to three tumours, < 3 cm each) are generally considered curable [3]. Curative treatment options include thermal ablation (TA, which includes microwave ablation and radiofrequency ablation), hepatic resection (HR), and orthotopic liver transplantation (OLTx) [4]. The latter is generally considered to yield the best recurrence-free survival [5], but is limited by a scarcity in available donor organs, and considerable procedural morbidity and mortality [6]^.^ Conversely, liver-sparing treatment modalities are plagued by higher rates of tumour recurrence [3]. Typically, initial therapy is with either HR or TA, after which some patients may eventually undergo OLTx [7].

Controversy still shrouds the optimal treatment strategy for early and very early HCC [8]. It has been shown that carefully performed TA can result in excellent locoregional control of primary HCC [9]. Data on outcomes after repeat TA for recurrence following succesful primary TA, however, is sparse. Even more controversial is the timing of OLTx after successful TA in patients with preserved liver function [8]. Should TA be considered ‘only’ as a stop-gap measure until curative treatment is performed in the form of OLTx, or can TA itself, in selected cases, be considered a definitive therapy, provided that strict follow-up imaging is performed, in order to detect possible recurrences at an early stage? We aim to contribute to this debate by presenting our data on outcomes after primary and repeat TA, with and without subsequent OLTx, in a large tertiary referral centre.

## Patients and methods

The present study focuses on outcomes after primary and repeat TA for HCC, with and without subsequent OLTx. In our centre, all cases of HCC are discussed in a multidisciplinary team. From 2000 onward, percutaneous TA was performed in a joint effort by specialised interventional radiologists and hepatobiliary surgeons. All patients who underwent TA, HR, or OLTx for HCC were prospectively entered into a database. In this study, we analysed patients who underwent primary TA between January 2000 and December 2019. Follow-up was completed up to December 2020. Outcomes studied were recurrence rate after primary TA and after its repeat interventions, the occurrence of untreatable recurrence, OS and DSS after primary TA and repeat interventions, and complications after TA (graded according to Clavien-Dindo [10]). We reconstructed patient-specific treatment paths, registering treatment modalities, time of recurrence (if any), and follow-up status. In order to compare survival without bias from tumour stage, age or comorbidity, we analysed patients who had been waitlisted for OLTx at any time after primary TA (*“intention-to-perform-OLTx”*), and compared them to a cohort of patients matched for age (< 70 years), tumour size and number (within Milan criteria) and comorbidity (ASA < 3; no severe cardiopulmonary comorbidity), who upon revision of charts by a liver transplant surgeon would have been eligible for OLTx from a medical point of view, but who were never waitlisted due to other reasons (*“eligible, non-waitlisted”*).

All instances of percutaneous TA were performed under computer tomography (CT) guidance while patients were under general anesthesia, and mechanically ventilated. Image acquisition and needle positioning were performed under controlled apnea during an expiratory breath hold, allowing for constant anatomical relations. We believe this to be critical to our TA workflow, as accurate needle positioning is a prerequisite of curative locoregional treatment [11]. All patients underwent a contrast-enhanced CT scan one week after TA, to assess completeness of ablation and to obtain a baseline scan for optimal comparison during follow-up. If this scan revealed an inadequate ablation zone, patients underwent completion ablation as soon as possible. This was defined as incomplete ablation, and the completion ablation was not counted as a separate procedure, in accordance with the standardised reporting criteria proposed by Ahmed et al. [12]. Follow-up consisted of 4-monthly contrast-enhanced CT scans, laboratory workup, including serum alpha-foetoprotein (AFP) and, where indicated, MRI or other imaging studies. We defined recurrence as any lesion arising after successful treatment (as evidenced by satisfactory margins on the CT scan at day 7), either with the radiological characteristics of HCC, or based on histopathological examination. Time of recurrence was defined as the timepoint at which any such lesion was first detected. Where missing, dates and causes of death were registered by contacting patients’ general practitioners. In case of doubt, death was assumed to be due to HCC. Survival was calculated separately from the date of primary intervention, and from the date of any subsequent interventions. This eliminates the time span between interventions, which might erroneously be interpreted as an increase in survival when multiple interventions have been performed (comparable to “lead-time” bias). Recurrence-free survival (RFS) was calculated from the time of intervention to recurrence of HCC; DSS was calculated from the time of intervention to the time of death from HCC.

Statistical analysis was performed with IBM SPSS (version 23). A χ^2^ test was used for categorical variables. Means or medians were compared with a Student’s T-test, or with a nonparametric test, as appropriate. Hazard ratios for all-cause mortality were calculated with a Cox regression model. Survival was calculated with a Kaplan–Meier model, and groups were compared with logostic regression. The threshold for statistical significance was set at *p* < 0·05.

## Results

### Baseline characteristics

In our centre, 330 patients underwent a total of 480 interventions with curative intent for HCC. Patients who underwent primary HR, combined HR with intraoperative TA, or OLTx as their primary interventions (n = 154) were excluded. The remaining 176 patients underwent primary TA: RFA in 72 patients (40·9%), and MWA in 104 patients (59·1%) Date of primary TA was January 2015 or later in 52·2% of cases, and January 2017 or later in 34·7% of cases. Table 1 shows baseline characteristics. Over 90% of patients had cirrhosis. Steatohepatitis was the most frequent cause of liver disease, followed by viral hepatitis. At presentation, patients had one to four detectable tumours on pre-operative imaging. At presentation, 50 patients had multiple tumours (28·4%). Median tumour size was 2·7 cm; a quarter of patients had at least one tumour ≥ 3·3 cm in diameter.

**Table 1:** Patient and tumour characteristics at time of primary thermal ablation (TA)

Demographics	Patients (*N* = *176)*
Median age (IQR; range)	64·5 years; (58·0–70·8; 12-90)
Male sex, N (%)	142 (80·7%)
Cirrhosis	
Yes	159 (90·3%)
Child–Pugh class	
A	151 (89·3%)
B	18 (10·7%)
Primary Underlying Liver Disease	
(N)ASH	94 (53·4%)
Viral	53 (30·1%)
Metabolic	6 (3·4%)
Cholestatic	5 (2·8%)
Cryptogenic cirrhosis	8 (4·5%)
Other/unknown	10 (5·7%)
Tumour characteristics	
Median diameter of largest tumour (IQR; range)	2·7 cm (2·0–3·3; 0.5-8.0)
Number of tumours	
1	126 (71·6%)
2	42 (23·9%)
3	6 (3·4%)
4	2 (1·1%)
BCLC stage	
Very early	22 (12·5%)
Early	136 (77·3%)
Intermediate	18 (10·2%)
Type of TA	
RFA	72 (40.9%)
MWA	104 (59.1%)
AFP level	
Median (IQR; range)	7·8 (3·6–27·0; 1–54,000)

### Recurrence and repeat interventions after primary TA

**Fig. 1:**
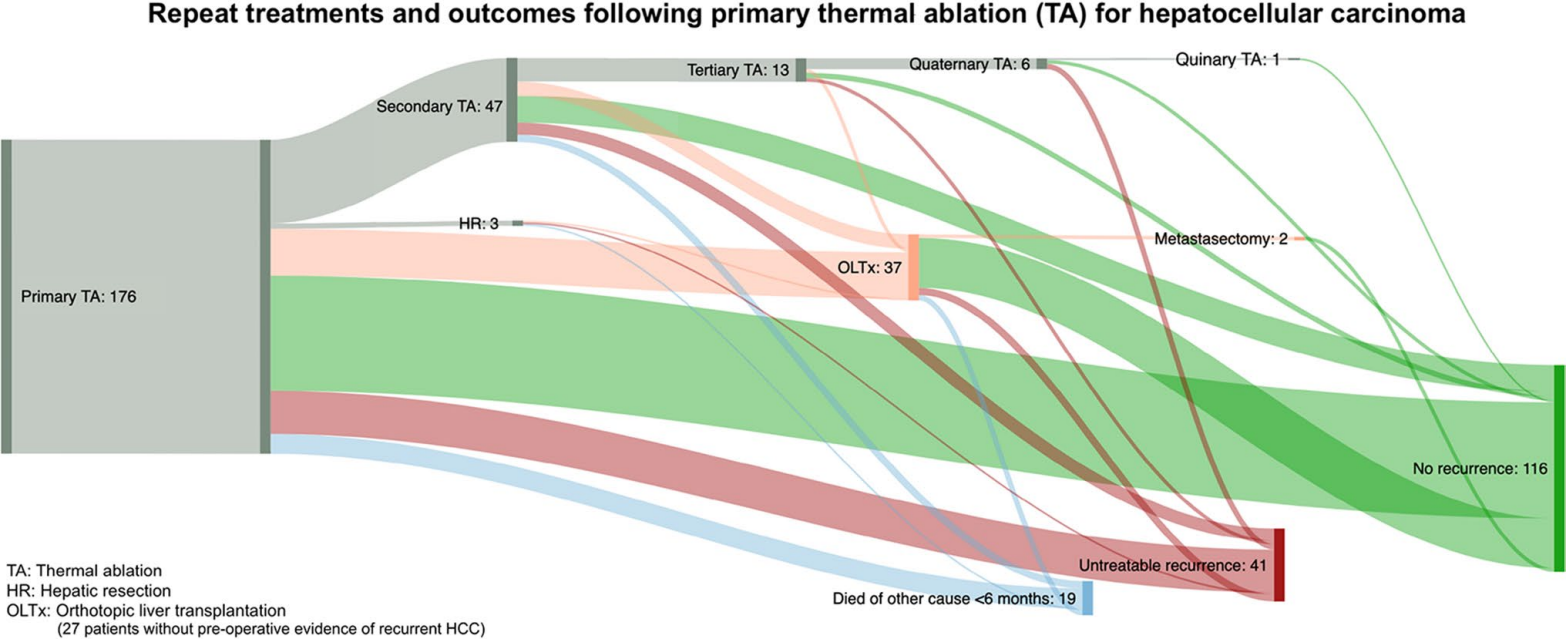
Patient-specific treatment paths following primary thermal ablation (TA) for HCC. The order of, and outcomes after subsequent re-interventions are shown. More highly detailed, fully quantitative data can be found in the flow chart in Supplementary Materials

After primary TA, routine CT on day 7 showed satisfactory ablation margins in 161 patients (91·5%). The remaining 15 patients (8·5%) all underwent successful completion TA, which were not counted as repeat ablations [12]. Median follow-up was 30·9 months (IQR 13·4—66·0). During follow-up, recurrences were detected in 99 of 176 patients (56·3%), of which 85 were intrahepatic (85·9%). In patients who were treated with repeat TA for recurrence after primary TA, the median time to recurrence was 13 months (IQR 6–23 months). Patient-specific treatment paths and outcomes are shown in Fig. 1 (for more details, fully quantitative data can be found in the Supplementary Material). Of 85 patients with intrahepatic recurrence, 76 patients (89·4%) underwent between 1 to 4 repeat interventions (TA, HR, OLTx) with curative intent. Not counting 19 patients (10·8%) who never had the chance to develop recurrent HCC due to death from other causes within 6 months of their last intervention, 116 of 176 patients (65·9%) remained recurrence-free during a median follow-up of 27·5 months after their last intervention. At any point in time after primary TA, a total of 41 (23·3%) patients developed recurrences that could not be treated with repeat TA, HR or OLTx. In this group, 21 patients (51·2%) underwent transarterial chemoembolisation (TACE) or selective intra-arterial radiotherapy (SIRT) for locally advanced disease, 13 patients (31·7%) were treated with sorafenib for metastatic disease, and the remaining 7 patients (17·1%) received best supportive care. A total of 37 patients (21·0%) underwent OLTx at any point in time after primary TA; and three patients (1·7%) underwent HR. In two patients (1·1%), both after OLTx following primary TA, resection of a late, solitary distant metastasis was performed, after which they remained recurrence-free. Excluding 37 patients who underwent OLTx after one or more sessions of TA, 139 “TA-only” patients remained. Not counting 16 patients (11·5%) with non-HCC-related death within 6 months after their last intervention, 86 of 139 TA-only patients (61·9%) remained recurrence-free following their last intervention. Eventually, 37 of 139 patients (26·6%) developed incurable recurrence.

### Survival

At the end of follow-up, 65 patients (36.9%) had died. Causes of death were HCC in 27 cases, (41.5% of deaths), progression of liver disease without signs of recurrent HCC in 11 cases (16.9%), and other causes in 27 cases (41.5%). Median OS after primary TA was 96·0 months (95% CI 41·0–150·9). At 5 years, OS was 54·0%. Median RFS after TA was 19·0 months. Median DSS was not reached. At 5 years, DSS was 73·8%. Table 2 shows hazard ratios for death for different patient and tumour related factors. Age, number of tumours and AFP levels were associated with mortality.

**Table 2:** Correlations of different clinical features with all-cause mortality

	Univariate		Multivariate	
	HR	*p*	HR	*p*
Age	1·029	**0·036**	1·029	**0·063**
Cirrhosis	2·113	0·207	N/A	N/A
HBV	0·743	0·369	N/A	N/A
HCV	0·840	0·566	N/A	N/A
Alcohol abuse	1·075	0·258	N/A	N/A
MELD score	1·077	0·121	N/A	N/A
*N* of tumours at baseline	1·492	**0·020**	1·497	**0·041**
Baseline tumour diameter	1·020	0·166	N/A	N/A
aFP level at baseline	1·001	** < 0·001**	1·001	** < 0·001**
MWA (vs. RFA)	1·050	0·848	N/A	N/A

Bold emphasis on *P* values reaching or approaching statistical significance

### Primary TA *versus* repeat TA

**Fig. 2:**
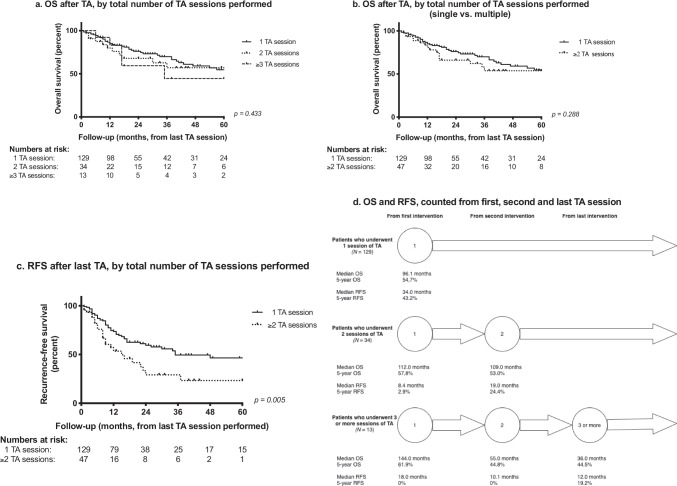
A. Overall survival, calculated from the last session of thermal ablation (TA) performed, stratified by total number of TA sessions performed(1, 2 or ≥ 3). B. Overall survival, calculated from the last session of TA performed, comparing those patients who underwent only primary TA to patients who underwent ≥ 2 sessions of TA. C. Recurrence-free survival, calculated from the last session of TA performed, comparing those patients who underwent only primary TA to patients who underwent ≥ 2 sessions of TA. D. Overall and recurrence-free survival, calculated from the date of primary TA (1), from the first session of repeat TA (2), and from the last session of repeat TA (3 or more). Values are shown separately for patients who underwent one, two, or more sessions of TA

After primary TA, 49 of 176 patients (27·8%) underwent up to four sessions of repeat TA, resulting in a total of 243 ablation sessions. During 67 sessions of repeat TA, patients were treated for solitary tumours in 52 sessions (77·6%, versus 71·6% at primary TA; *p* = 0·218). Mean tumour diameter during repeat TA (2·8 cm; SD 1·3, range 0·8–3·2) was comparable to mean tumour diameter at primary TA (2·9 cm; SD 1·2; range 0·5–8·0; *p* = 0·810). Figure 2 shows OS and RFS after primary TA, after one session of repeat TA, and after multiple sessions of repeat TA. We found no statistically significant differences in OS calculated from the date of the last-performed TA session, neither when comparing between all three groups separately (primary TA only; one session of repeat TA; two or more sessions of repeat TA: *p* = 0·433, Fig. 2A), nor when combining all patients with repeat TA into one group (*p* = 0·288, Fig. 2B). RFS, however, was shorter after repeat TA, compared to patients who underwent only primary TA (*p* = 0·005, Fig. 2C). Quantitative survival times, calculated from primary and repeat interventions, are indicated in Fig. 2D.

Incidence and severity of complications (graded according to Clavien-Dindo [10]) were comparable following primary and repeat TA sessions (p = 0·840). After primary TA, there were no complications in 132 patients (75·0%), grade 1–2 complications in 33 patients (18·8%), and complications graded 3 and above in 11 patients (6·3%). After 67 sessions of repeat TA, there were no complications following 56 sessions (83·6%), grade 1–2 complications following 10 sessions (14·9%), and a grade 3 complication following 1 session (1·5%). TA-related 30-day mortality was 1.2%.

### Primary TA followed by OLTx *versus* no subsequent OLTx

**Fig. 3:**
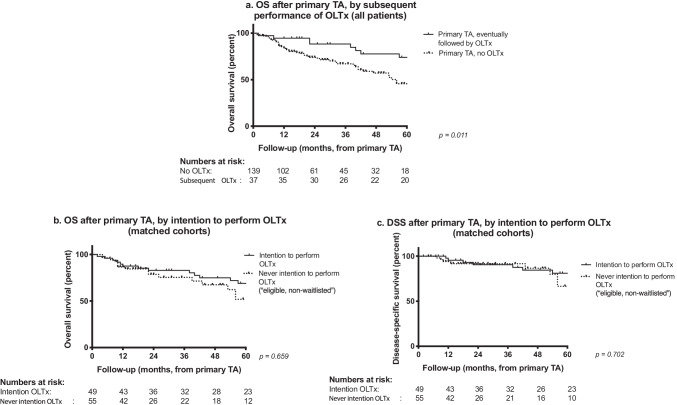
A. Overall survival calculated from the date of primary thermal ablation (TA), comparing all patients who subsequently underwent orthotopic liver transplantation (OLTx) to those who did not. B. Overall survival calculated from the date of primary TA, comparing those patients who were actively waitlisted for OLTx (“intention-to-perform OLTx") to a matched cohort of patients who would have qualified OLTx, but were not waitlisted due to either patient or physician preference, or ongoing alcohol abuse (“eligible, non-waitlisted”). C. Disease-specific survival calculated from the date of primary TA, comparing the same matched groups as in B

After primary TA (*n* = 176), 55 patients were screened and accepted for the OLTx waitlist. After having been accepted, six patients refrained from active waitlisting due to various non-medical reasons, leaving 49 patients who were awaiting a donor liver at any point in time. Reasons not to screen patients for OLTx were age > 70 years in 53 cases (of 121 non-waitlisted patients, 43·8%), unfavourable oncology in 10 (8·3%), prohibitive comorbidity in 9 (7·4%), patient or physician preference in 25 (20·7%), ongoing alcohol abuse in 8 (6·6%), and other reasons in 16 (13·2%). Eventually, 37 of 49 actively waitlisted patients (75·5%) underwent OLTx. There was no pre-operative evidence of recurrent or residual HCC before OLTx in 27 patients (73.0%). A comparison of clinicopathological characteristics of transplanted versus non-transplanted patients is shown in Table 3. Transplanted patients were younger, more often male, HCV positive and universally cirrhotic. Survival for both groups is shown in Fig. 3. Median and 5-year OS were better for patients who underwent OLTx (137 vs. 54 months; 74·7% vs. 45·0%; *p* = 0·011*,* Fig. 3A). To correct for both lead-time bias due to the interval between TA and OLTx, and selection bias due to higher age, more severe comorbidity, and more unfavourable oncology for the non-OLTx group, we calculated survival for an “*intention-to-perform-OLTx” group,* consisting of all patients who had at any point in time been actively waitlisted with the intention to perform OLTx after primary TA (*n* = 49), and compared them to an *“eligible, non-waitlisted”* group (*n* = 55). This latter group consisted of those patients who would have qualified for OLTx on medical grounds (see methods), but were never actively waitlisted due to other reasons. Groups had comparable median age (p = 0.835), Child–Pugh Class (p = 0.305), AFP (p = 0.840), tumour size (p = 0.426), and number of tumours (p = 0.724) at primary TA. OS was comparable between these groups (median 121·6 months vs. 109·8 months; 5-year OS 68·7% vs. 52·9%; *p* = 0·659, Fig. 3B), as was DSS (81·1% vs. 66·7% at 5 years, *p* = 0·702, Fig. 3C).

**Table 3:** Clinicopathological characteristics of patients who underwent orthotopic liver transplantation (OLTx) after primary thermal ablation (TA), versus those who did not

Parameters at time of primary TA	Never OLTx (*n* = 139)	Eventual OLTx (*n* = 37)	
Median age (IQR)	66·5 (59·0–71·3)	59·5 (53·7–65·0)	***p* = *0·002***
Male sex	107 (77·0%)	35 (94·6%)	***p* = *0·031***
Median diameter of primary tumour (IQR)	2·7 cm (2·1–3·3)	2·8 cm (2·0–3·6)	*p* = *0·344*
Number of tumours at primary intervention			*p* = *0·304*
*1*	97 (69·8%)	29 (78·4%)	
*2 or more*	42 (30·2%)	8 (21·6%)	
Cirrhosis	122 (87·8%)	37 (100%)	***p* = *0·018***
Child–Pugh B	13 (9·7%; 5 missing)	5 (14·3%; 2 missing)	*p* = *0·652*
HCV	19 (13·7%)	13 (35·1%)	***p* = *0·006***
Median AFP at baseline	7·2	7·9	*p* = *0·864*

Bold emphasis on *P* values reaching or approaching statistical significance

## Discussion

We found TA to be a reliable, repeatable treatment modality for HCC. After primary TA, almost half of patients never developed clinically apparent recurrence during follow-up. Most recurrences could be treated with curative intent, either with repeat TA or with OLTx. Some patients underwent numerous sessions of TA without developing an incurable recurrence. Even though RFS was shorter after repeat TA than after primary TA, we found no difference in OS. In fact, after matching patients for baseline oncological characteristics, comorbidity, and age, we found that OS after TA without ever being waitlisted for OLTx was comparable to OS after TA as a step-up to OLTx. Our findings imply the existence of a group of patients who do remarkably well with TA alone, some despite developing multiple recurrences. There is almost no data on outcomes after repeated sessions of TA. A literature search on PubMed-listed studies from January 2015 to 2021 (search terms: ((HCC[Title]) OR (hepatocellular carcinoma[Title])) AND ((ablation[Title]) OR (RFA[Title])) AND ((redo[Title]) OR (recurrence[Title]) OR (repeat[Title]) OR (recurrent[Title])), yielded 104 hits. Manual screening revealed two studies which reported on survival after repeat TA following primary TA [13, 14]. Both were performed in Asian populations, with comparatively young patients (median age 50 [13], and 60 [14]), with almost exclusively viral hepatitis-induced HCC. One study reported a number of non-cirrhotic patients above 30% [13]. Therefore, we believe these studies are not generalisable outside of their highly specific demographic contexts.

We found a median OS after primary TA of 96.0 months, and a 5-year OS of 54·1%. A widely varying 5-year OS after TA has been reported in the literature, mostly around 40–60% [15–17], (outliers in selected populations 30%-80% [18–20]). It must be noted that our patient population is relatively old (median 64·5 years; 25% of patients ≥ 70 years), while most studies had a median patient age around 55 years [17, 20], with only sporadic studies in a comparable age group [16, 19]. Furthermore, tumours in our study were relatively large (median 2·7 cm, 25% of tumours ≥ 3·3 cm), whereas many studies excluded tumours > 3 cm [15, 21], or had such tumours only in small numbers [20]. Lastly, some authors excluded patients with multiple tumours [21] or neo-adjuvant therapy [19].

As causes of death are variable in patients with chronic liver disease, DSS may be a better measure of oncologic results, especially when comparing results across different populations. Unfortunately, few studies report DSS. Our 5-year DSS of 73·8% after TA is high compared to the literature [22, 23].

Our finding that 13·6% of patients developed incurable recurrence following primary TA (24% of first recurrences) appears to be in contrast to the suggestion by Doyle et al. that 42% of recurrences are beyond Milan criteria, when analysing a series of smaller, unifocal primary HCC’s [21]. Despite some patients undergoing up to four sessions of repeat TA, only 23·3% of our patients eventually developed a recurrence unamenable to repeat TA or OLTx. These results might be because of the relatively high technical success rate of our TA workflow, and due to our strict follow-up protocol, which allows us to detect recurrences early on.

Over the past two decades, TA has established itself as an effective treatment option for HCC. Little evidence supports its formerly assumed inferiority to HR [19, 24]. Indeed, our DSS after TA is not inferior to rates reported after HR [25, 26]. More uncertain is the optimal relationship between TA and OLTx. On the one hand, OLTx leads to the best recurrence-free survival [5], with some authors proclaiming OLTx to be the only curative treatment [27], and others suggesting that locoregional treatments for HCC should be thought of as “a bridge to nowhere” [28]. By removing the preneoplastic cirrhotic liver parenchyma in its entirety, OLTx adresses the driving factor behind HCC recurrence [25]. On the other hand, OLTx is a major procedure with a lasting impact on patients’ lives [6], and is restricted by a shortage of donor organs. It is therefore imperative that we put these scarce resources to optimal use.

Our findings support a more nuanced view of the role of TA in early and very early HCC than hitherto proposed by many. Until now, clinicians have focused on identifying patients who have the lowest risk of post-transplant recurrence [29], but this is only one side of the picture. Ideally, we should reserve OLTx for patients who would have bad outcomes with locoregional therapy alone [30].

A shortcoming of our study is its observational nature. This brings with it an inherent danger of confounding variables and selection bias, for which it is impossible to fully correct. Furthermore, in some subgroup analyses, smaller numbers may have obscured underlying correlations. The heterogeneity of this patient population makes a sound prospective analysis beyond the first treatment modality extremely difficult.

In conclusion, we found that TA is an effective treatment modality both for primary and recurrent HCC. Although many patients develop recurrences during follow-up, we demonstrated that most can be treated with repeat TA. There is a group of patients with HCC who survive many years with TA, without ever undergoing OLTx. In matched cohorts, TA without waitlisting for OLTx was not inferior to TA with the intention to perform OLTx. Therefore, it is imperative that we find a way to differentiate between those patients who would do well with TA alone, and those who will require OLTx due to a high risk of untreatable tumour recurrence. This way, we can put scarce donor organs to best use. It is beyond any doubt that if a “TA-first” approach is taken, intensive follow-up is crucial.

## Supplementary Information

Below is the link to the electronic supplementary material.

Supp. 1. Fully quantitative, patient-specific treatment paths following primary thermal ablation (TA) for HCC. The order of, and outcomes after subsequent re-interventions are shown. OLTx: orthotopic liver transplantation; HR: hepatic resection. Supplementary file1 (PDF 97 kb)

## Abbreviations

AFP: Alpha-foetoprotein
CT: Computed tomography
DSS: Disease-specific survival
HCC: Hepatocellular carcinoma
HR: Hepatic resection
OLTx: Orthotopic liver transplantation
OS: Overall survival
RFS: Recurrence-free survival
TA: Thermal ablation

## Declarations

### Guarantor

The scientific guarantor of this publication is K.P. de Jong, Hepato-Pancreato-Biliary surgeon, transplant surgeon, and head of the locoregional treatment team of the Department of Hepato-Pancreato-Biliary Surgery and Liver Transplantation at the University of Groningen.

### Statistics and Biometry

No complex statistical methods were necessary for this paper.

### Methodology

• retrospective

• observational

• performed at one institution
