# Supplementary material for: Outcomes after primary and repeat thermal ablation of hepatocellular carcinoma with or without liver transplantation
Source: Eur Radiol. 2022 Feb 8;32(6):4168–76. doi: 10.1007/s00330-021-08515-3 (PMC9123025; doi:10.1007/s00330-021-08515-3)
Supplement: Supplementary file 1 — Supp. 1. Fully quantitative, patient-specific treatment paths following primary thermal ablation (TA) for HCC. The order of, and outcomes after subsequent re-interventions are shown. OLTx: orthotopic liver transplantation; HR: hepatic resection. Supplementary file1 (PDF 97 kb) [file 330_2021_8515_MOESM1_ESM.pdf]

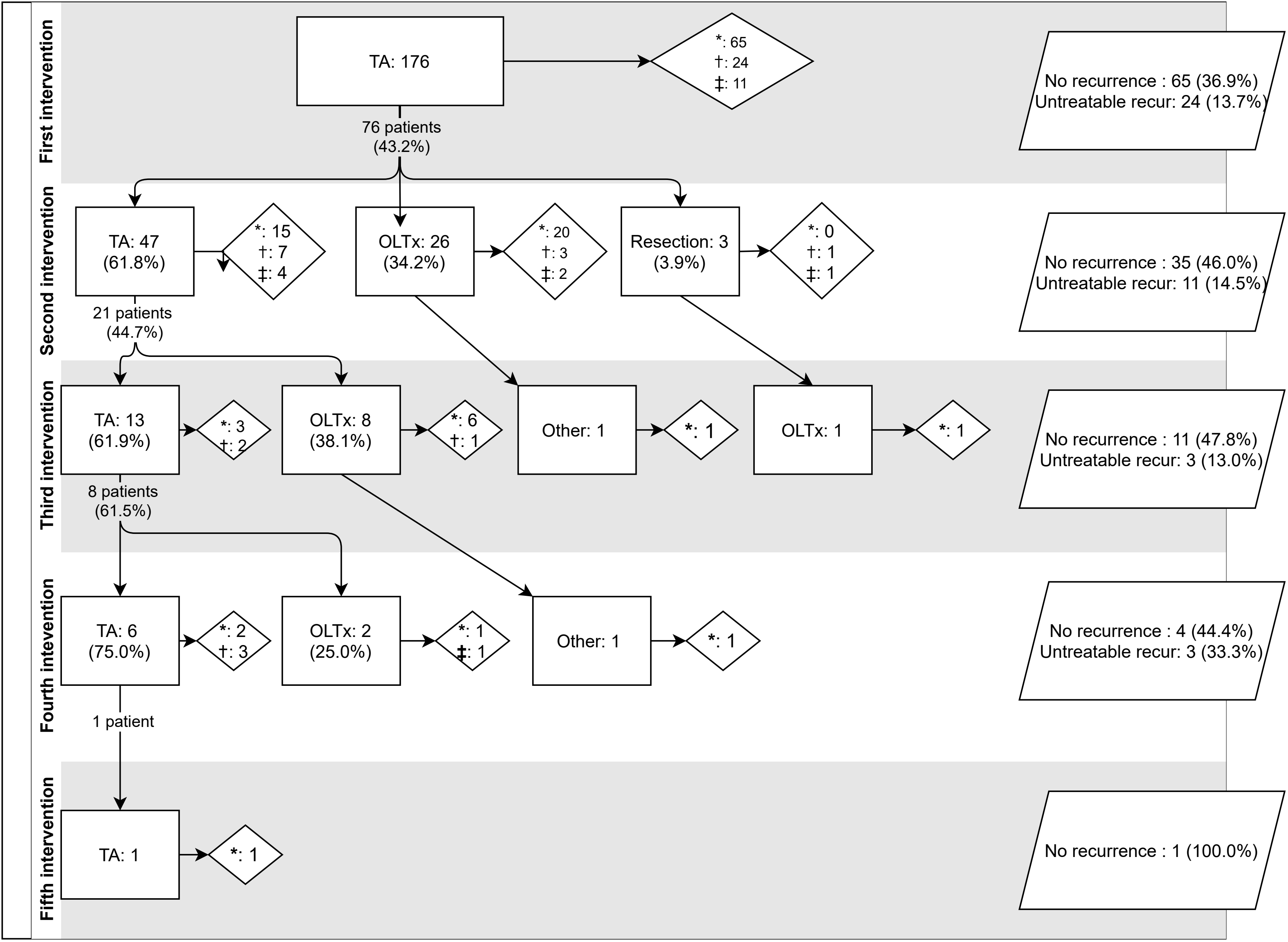

*: No recurrence detected during follow-up

†: Untreatable recurrence, no curative options

‡: Died from non-HCC cause <6 months
